# First-Principles Calculations of Angular and Strain Dependence on Effective Masses of Two-Dimensional Phosphorene Analogues (Monolayer *α*-Phase Group-IV Monochalcogenides *MX*)

**DOI:** 10.3390/molecules24030639

**Published:** 2019-02-12

**Authors:** Yuanfeng Xu, Ke Xu, Hao Zhang

**Affiliations:** 1 School of Science, Shandong Jianzhu University, Jinan 250101, China; 2 Key Laboratory for Information Science of Electromagnetic Waves (MoE), Key Laboratory of Micro and Nano Photonic Structures (MoE) and Department of Optical Science and Engineering, Fudan University, Shanghai 200433, China; 18210720004@fudan.edu.cn

**Keywords:** first-principles calculations, phosphorene analogues, effective mass, anisotropic property

## Abstract

Group IV monochalcogenides MX (*M* = Ge, Sn; *X* = S, Se)-semiconductor isostructure to black phosphorene-have recently emerged as promising two-dimensional materials for ultrathin-film photovoltaic applications owing to the fascinating electronic and optical properties. Herein, using first-principles calculations, we systematically investigate the orbital contribution electronic properties, angular and strain dependence on the carrier effective masses of monolayer MX. Based on analysis on the orbital-projected band structure, the VBMs are found to be dominantly contributed from the $p_{z}$ orbital of *X* atom, while the CBM is mainly dominated by $p_{x}$ or $p_{y}$ orbital of *M* atom. 2D SnS has the largest anisotropy ratio due to the lacking of *s* orbital contribution which increases the anisotropy. Moreover, the electron/hole effective masses along the *x* direction have the steeper tendency of increase under the uniaxial tensile strain compared to those along *y* direction.

## 1. Introduction

Two-dimensional (2D) materials have invoked enormous research interests due to the unique properties that may lead to promising applications in various fields of nanoelectronics, optoelectronics and thermoelectronics [1,2,3,4,5,6]. Recently, monolayer black phosphorene [7], with puckered low symmetry structure, was introduced in the 2D materials family with largely tunable direct bandgap (thickness dependence) suitable for high-speed photodevice [8,9], high carrier transport property comparable to that of graphene [10,11], and strong in-plane anisotropy probably leading to promising applications in polarization sensitive photodetector [12,13,14]. Monolayer group-IV monochalcogenides MX (*M* = Ge, Sn; *X* = S, Se), obtained by mutating two P atoms in black phosphorene by group IV-VI elements, called 2D “phosphorene analogues”, also possess relatively high carrier mobility and strong in-plane anisotropy [15] as well as other interesting properties in optics [16,17], piezoelectricity [18,19], thermoelectric and phonon transport properties [20].

Generally, the lattice mismatch between 2D materials and substrates during the process of experimental realization, often results in the formation of crystal distortion [21,22,23,24], which usually can be regarded as the results of applying strains. Furthermore, for anisotropic 2D materials like MX studied here, the physical properties measured in experiments depend on measurement angles [25,26]. Therefore, investigations on the angular and strain dependence of properties of monolayer group IV monochalcogenides MX are thus necessary to gain insight into the underlying mechanisms.

In this work, we perform first-principles calculations based on the density functional theory (DFT) to analyze the orbital-projected electronic band structure, angular and strain dependence on the carrier effective masses of group IV monochalcogenides MX. We demonstrate that the fundamental band gaps are indirect for monolayer GeS, SnS, SnSe, and direct for monolayer GeSe. The calculated band gap by Heyd-Scuseria-Ernzerhof (HSE) method is 2.75 eV for SnSe which is feasible to tune semiconductor property from indirect to direct, and makes it promising for the application of optoelectronic devices working within visible spectrum. Furthermore, we also investigate the angular dependence and strain engineering on the carrier effective mass of monolayer MX.

## 2. Method and Computational Details

The calculations are performed using the Vienna Ab Initio simulation package (VASP) based on density functional theory (DFT) [27]. The exchange-correlation energy is described by the generalized gradient approximation using the Perdew-Burke-Ernzerh (PBE) of functional [28], and the effect of the van der Waals interactions between adjacent layers is considered by using the empirical correction scheme of Grimme’s DFT-D2 (PBE-D2) method [29,30]. The calculation is carried out by using the projector-augmented-wave (PAW) pseudopotential method with a plane-wave basis set with a kinetic energy cutoff of 500 eV. Furthermore, for Ge, Sn and S, Se atoms, we considered *d* and *p* semicore states, respectively, as valence states by choosing Ge-d, Sn-d and S, Se pseudopotentials. When optimizing atomic positions, the energy convergence value between two consecutive steps are chosen to be 10${}^{-6}$ eV and the maximum Hellmann-Feynman (HF) force acting on each atom is 10${}^{-3}$ eV/Å. The Monkhorst-Pack scheme are performed for the Brillouin zone integration with *k*-point meshes of 13 × 11 × 1 and 17 × 15 × 1 for geometry optimization and self-consistent electronic structure calculations, respectively. To verify the results of the PBE calculations, the electronic structures of monolayer MX are calculated using the much more computationally expensive hybrid HSE06 functional [31,32]. Generally, HSE06 improves the precision of band gap by reducing the localization and delocalization errors of PBE and HF functionals. Hereby, the screening parameter u is set to 0.2 Å${}^{-1}$.

The effective masses $m^{*}$ of monolayer MX along the transport direction for holes and electrons, are obtained by fitting parabolic functions to the valence-band maximum (VBM) and conduction-band minimum (CBM), respectively, given by,

(1) $$m^{*}=\hbar^{2}({\partial^{2}E(k)/\partial k^{2})}^{-1},$$

where *ℏ* is the reduced Planck’s constant, *k* is wave-vector, and E(k) denotes the energy.

## 3. Results and Discussions

### 3.1. Optimized Structures of Monolayer MX (M = Ge, Sn; X = S, Se)

**Figure 1:**
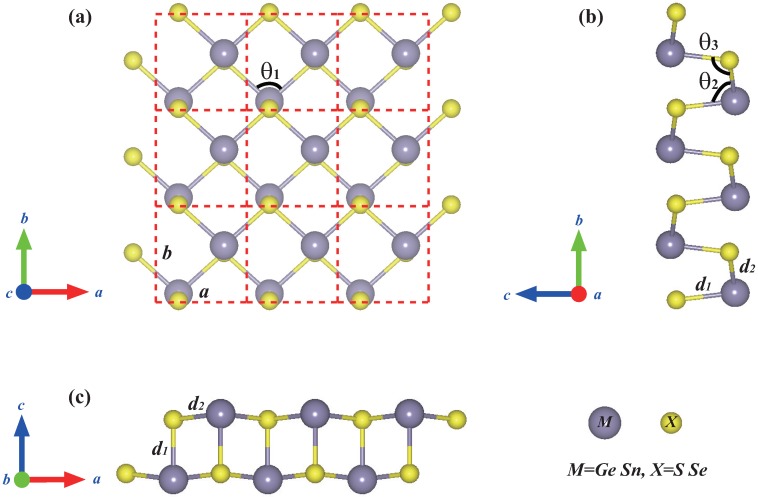
Atomic structure of group IV monochalcogenides MX in a 3 × 3 × 1 supercell from (**a**) top view and (**b**,**c**) side views.

The monolayer group-IV monochalcogenides MX are formed by the covalent bonding between group-IV (*M* = Ge, Sn) elements and group-VI (*X* = S, Se) elements, as shown in Figure 1. They have puckered atomic arrangement similar to that of black phosphorene, which possesses an orthorhombic crystal structure with the space group of $Pmn2_{1}\left(31\right)$. The unit cell (the red dashed rectangle) of monolayer MX contains two *M* atoms and two *X* atoms. The optimized lattice parameters and geometrical properties of monolayer MX are summarized in Table 1. For example, the calculated lattice constants of monolayer GeSe are *a* = 3.935 Å and *b* = 4.248 Å, which are in good accordance with previous experimental (*a* = 3.83 Å and *b* = 4.39 Å) [33] and theoretical results (*a* = 3.93 Å and *b* = 4.27 Å) [18]. Each layer of GeSe consists of buckled six-membered rings (e.g., “six-rings”; three Ge atoms and three Se atoms) in a so-called chair conformation. This “six-rings” structure has two vertical Ge-Se bonds with bond length of $d_{1}$ = 2.529 Å and four lateral Ge-Se bonds with bond length of $d_{2}$ = 2.642 Å. Bulk GeSe has a tetragonal unit cell with lattice parameters *a* = 3.833 Å, *b* = 4.394 Å and *c* = 10.840 Å, belonging to Pbnm space group [34]. A small difference exists in the lattice constants (*a* and *b*) between monolayer and bulk GeSe, which can be attributed to the consequence of interlayer van der Waals interactions in few-layer and bulk GeSe.

The optimized lattice parameters of other primitive cells (monolayer GeS, SnS and SnSe) are also listed in Table 1, which are in good agreement with previous theoretical results and experimental results [16,18,20,35,36,37,38]. Here, the structures are optimized with a large vacuum space of 20 Å in the *z* direction until the forces on each atom with different layers are close to zero. The *x* and *y* directions coincide with *a* and *b* directions, respectively, and the *z* direction is perpendicular to the xy plane.

### 3.2. Electronic Band Structures of Monolayer MX (M = Ge, Sn; X = S, Se)

**Figure 2:**
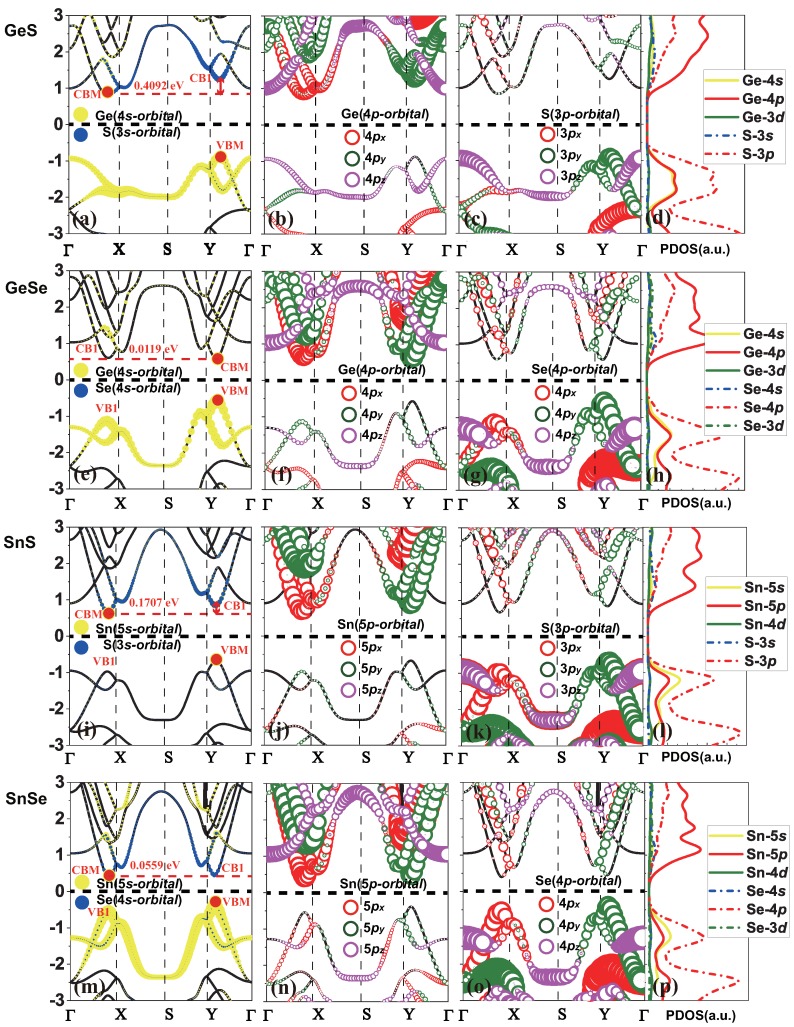
The site and orbital-projected band structure of monolayer GeS (**a**–**c**); GeSe (**e**–**g**); SnS (**i**–**k**) and SnSe (**m**–**o**). The yellow and blue circles represent *s* orbitals of *M* and *X* atoms, respectively. The red, green and purple circles represent $p_{x}$, $p_{y}$ and $p_{z}$ orbitals, respectively. Sample size is proportional to the weight of the atomic orbital in corresponding state. In addition, density of states (DOS) of monolayer GeS (**d**); GeSe (**h**); SnS (**l**) and SnSe (**p**). The black lines indicate the band structure. CB1/VB1 is another CBM/VBM with small energy difference from the real CBM/VBM.

The thermal and dynamical stability of monolayer group-IV monochalcogenides MX has been demonstrated [38,39,40,41], and here we systematically study the electronic band structures of monolayer MX, as shown in Figure 2 and Table 2. In order to give reliable results for the gaps, we have calculated the band structures of monolayer group-IV monochalcogenides MX under PBE functional and HSE06 hybrid functional, respectively. From the calculated band gaps shown in Table 2, it is found that monolayer GeS and GeSe possess band gaps ($E_{g}$) within the visible range, which makes them promising for applications of optoelectronic devices working in visible spectrum.

For a better understanding of the particular electronic features, the band structures are projected by the atomic orbitals of the *M* (Ge, Sn) and *X* (S, Se) atoms which are shown in Figure 2. From the partial density of states of *s*, *p* and *d* orbitals shown in Figure 2d,h,l,p, we found that *d* orbital nearly does not contribute to the total density of states near Fermi level, and the orbital contribution to band structures near Fermi level are dominated by *s* and *p* orbitals. In general, it is shown in Figure 2 that the lowest conduction bands of MX monolayers are dominated by M-p orbitals, while the highest valence bands are contributed from M-s, X-s and X-p orbitals, although the contribution from X-p orbitals is much larger than M-s and X-s orbitals.

Monolayer GeS is an indirect semiconductor with CBM located near the *X* point along the Γ-X direction, and the VBM locates near the *Y* point along the Γ-Y direction. The CBM is dominated by Ge-4$p_{x}$ orbital, while the VBM is dominated by S-3$p_{y}$ and Ge-4*s* orbitals. Monolayer GeSe is a direct semiconductor with CBM and VBM both located near the *Y* point along the Γ-Y direction. The CBM is dominated by Ge-4$p_{y}$ orbital, while the VBM is dominated by Se-4$p_{y}$ and Ge-4*s* orbitals. Monolayer SnS and SnSe are indirect semiconductors with the location of VBM and CBM similar with that of GeS. The CBM of SnS is dominated by Sn-5$p_{x}$ orbital, while the VBM is dominated by S-3$p_{y}$ orbital. The CBM of SnSe is dominated by Sn-5$p_{x}$ orbital, while the VBM is dominated by Se-4$p_{y}$ and Sn-5*s* orbitals. By comparison among atomic-orbital contributions for these four MX monolayers, it is found that VBM of SnS monolayer is different from the others, since the Sn-5*s* contribution is negligible to VBM. As we know, the *s* atomic orbital is non-dispersive and shows homogeneous in chemical bondings with other atoms, and the conduction/valence electronic states attributed to *s* orbitals are less anisotropic than those formed by p/d or hybrid orbitals, which finally would decrease the anisotropy in properties like effective masses. Furthermore, this phenomenon will be discussed in the next section.

It is worth mentioning that the energy differences ($E_{\Delta }$) between the local CBM (Γ-X) and CB1 (Γ-Y), i.e., 0.4092 eV, 0.0119 eV, 0.1707 eV, and 0.0559 eV, as listed in Table 2 for monolayer GeS, GeSe, SnS and SnSe respectively, are quite small, which manifest the existence of saddle-like points in the lowest conduction bands for monolayer MX. Similar saddle-points can be observed in the highest valence bands for monolayer MX, as shown in Figure 2. A remarkable light absorption caused by the formation of saddle-points excitons within the single-particle band gaps will be found in the monolayer MX due to the large enhancement of joint density of states and electron-hole interactions at the saddle points [44]. On the other hand, such small $E_{\Delta }$ between the two CBMs/VBMs along the Γ-X and Γ-Y directions in 2D MX indicate that the semiconductor properties may be tunable from direct to indirect or vise versa by the application of external controls (e.g., strain).

### 3.3. Angular Dependence of the Effective Mass of Monolayer MX (M = Ge, Sn; X = S, Se)

**Figure 3:**
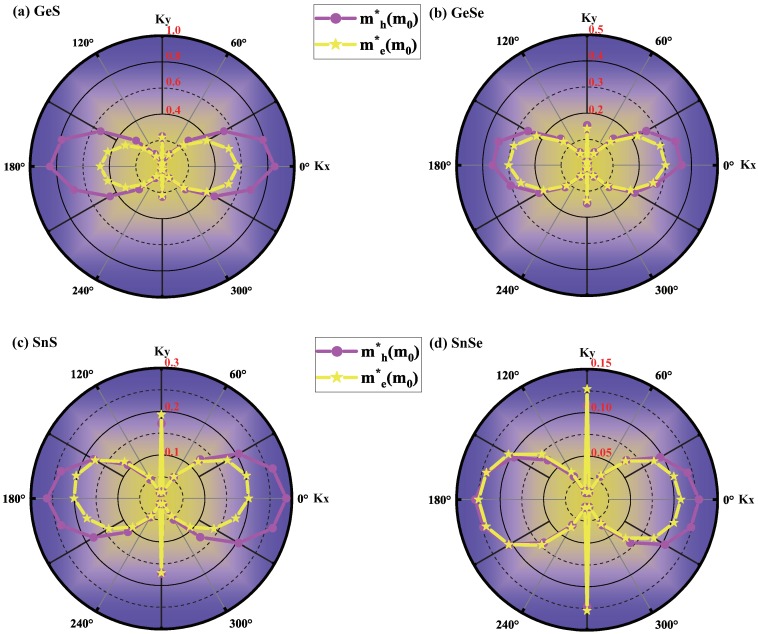
Angular dependence on the effective masses of holes and electrons of monolayer (**a**) GeS; (**b**) GeSe; (**c**) SnS and (**d**) SnSe. The purple and yellow lines indicate the effective mass curves of holes and electrons, respectively.

Electronic devices such as field effect transistors using 2D materials require light effective mass of mobile carriers in the xy plane and subsequent high carrier transport properties. The effective mass of carrier can be calculated from the high-precision electronic band calculation given by Equation (1). Table 2 only shows the effective mass along *x* and *y* direction. However, due to the anisotropic geometric structure, the carrier transport properties of monolayer MX are expected to be highly orientation-dependent. In order to get a full understanding of the carrier transport properties of monolayer MX, the angular-dependent effective masses are calculated as shown in Figure 3. In the polar plots of the obtained effective mass, the dots and the solid curves are the calculated values and fitting values, and the purple and yellow lines represent the effective mass of holes and electrons, respectively. With the ultralow spin-orbit-coupling (SOC) strength (around −15 meV) for monolayer MX, the SOC effect has been neglected in this work since it does not influence significantly the angular distribution of the effective masses.

The band structure of monolayer MX displays highly anisotropic dispersions near the VBM and CBM, leading to the anisotropic effective mass of holes and electrons along the Γ-X and Γ-Y directions as shown in Table 2. Furthermore, the 2D polar representation curves in Figure 3 clearly indicate that effective mass of monolayer MX is highly anisotropic. As listed in Table 2, for monolayer GeS, the calculated effective mass along Γ-X and Γ-Y are 0.850$m_{0}$(h)/0.572$m_{0}$(e) and 0.232$m_{0}$(h)/0.221$m_{0}$(e), respectively, which are in good agreement with previous results (Γ-X: 0.92$m_{0}$(h)/0.50$m_{0}$(e), Γ-Y: 0.23$m_{0}$(h)/0.22$m_{0}$(e)) [42]. The calculated effective mass ratios of hole $\gamma_{h}$ ($m_{h}^{x}$ divided by $m_{h}^{y}$) and electron $\gamma_{e}$ ($m_{e}^{x}$ divided by $m_{e}^{y}$) are 3.664 and 2.588, respectively, which show large anisotropy of effective mass when shifting the propagation direction from $0^{{}^{\circ}}$ (along x axis) to $90^{{}^{\circ}}$ (along y axis). For the rest of the three group IV monochalcogenides MX, the calculated carrier effective masses along the *x* and *y* directions are in good agreement with previous results as well [35,42,43].

For monolayer group IV monochalcogenides MX, the effective mass along the *x*-direction has the maximal value of 0.850$m_{0}$(h)/0.572$m_{0}$(e), 0.361$m_{0}$(h)/0.300$m_{0}$(e), 0.287$m_{0}$(h)/0.200$m_{0}$(e), and 0.128$m_{0}$(h)/0.108$m_{0}$(e) for GeS, GeSe, SnS, SnSe, respectively. The minimum value is 0.026$m_{0}$(h)/0.023$m_{0}$(e), 0.013$m_{0}$(h)/0.012$m_{0}$(e), 0.013$m_{0}$(h)/0.015$m_{0}$(e), and 0.008$m_{0}$(h)/0.009$m_{0}$(e) for GeS, GeSe, SnS, SnSe, respectively, which occurs along the direction of $75^{{}^{\circ}}+n\pi$, $105^{{}^{\circ}}+n\pi$ (*n* = 0.1). The effective mass decreases from GeS to SnSe, to SnS and to SnSe, which makes SnS and SnSe potential candidate for application in optoelctronic devices.

For monolayer GeSe, with the decrease of the values of the obtained effective mass along the Γ-X and Γ-Y directions, the effective mass ratios of hole $\gamma_{h}$ and electron $\gamma_{e}$ are 2.375 and 2.143, respectively, which shows smaller anisotropy of effective mass than that of GeS. For SnS and SnSe, the hole ratio $\gamma_{h}$ is 1.669 and 1.185, and the electron ratio $\gamma_{e}$ is 1.039 and 0.850, respectively, which are much smaller anisotropic than those of monolayer GeS and GeSe. Furthermore, by calculating the relative anisotrpy ratio with $\gamma_{h}/\gamma_{e}$, we investigate the relative anisotropy between VBM and CBM, which gives rise to a largest value of $\gamma_{h}/\gamma_{e}$ ($\gamma_{h}/\gamma_{e}=1.606$) for SnS monolayer among these four MX monolayers. The largest anisotropy ratio $\gamma_{h}/\gamma_{e}$ for SnS monolayer is caused by the increase of anisotropy of VBM due to lacking of *s* orbital contribution which has been discussed above.

### 3.4. Strain Dependence of Effective Mass of Monolayer MX (M = Ge, Sn; X = S, Se)

**Figure 4:**
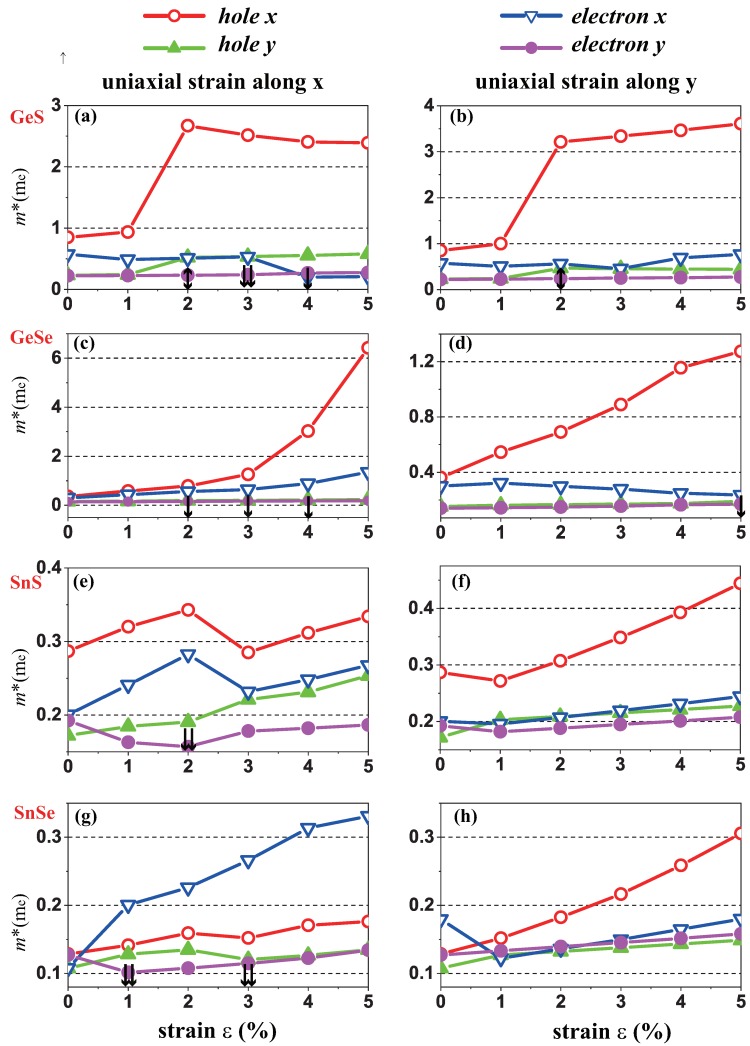
Evolution of carrier effective mass along the *x* and *y* directions of monolayer GeS, GeSe, SnS and SnSe under uniaxial strain along *x* (**a**,**c**,**e**,**g**) and *y* (**b**,**d**,**f**,**h**) directions, respectively.

Generally, the strain effect is due to the lattice mismatch between the substrate and 2D materials, or mechanical loading [45,46]. Indeed, the strain has a significant influence on the band structure of 2D materials [47,48], and the locations of VBM and CBM change accordingly. As shown in Figure 4, the symbols of ↕, ⇊ and ↓ represent the sharp change of VBM to Γ point, VBM/CBM→VB1/CB1, and the moderate change of CBM/VBM around the original location, respectively. The evolution of band structures under applied strains subsequently alters the effective mass of the carriers determined by the curvatures of the band edges near the Fermi level. Figure 4 demonstrates the evolution of the calculated effective masses of the electrons ($m_{e}^{*}$) and holes ($m_{h}^{*}$) along *x* and *y* directions under the uniaxial strain along *x* (Figure 4a,c,e,g) and *y* (Figure 4b,d,f,h) directions, and we constrained the tensile strain (ε) within the range from 0% to 5%. ε indicates the components of the relative strain along the transport directions. Table 3 shows the evolution range of the calculated carrier effective masses under tensile uniaxial strain.

**Figure 5:**
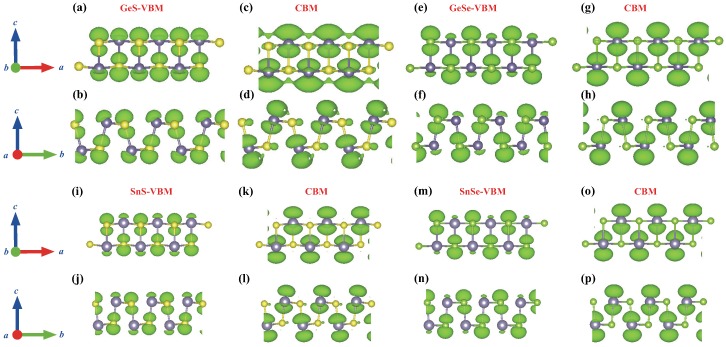
Isosurface plots of the charge density of VBM (**a**,**b**) and CBM (**c**,**d**) for monolayer GeS, VBM (**e**,**f**) and CBM (**g**,**h**) for monolayer GeSe, VBM (**i**,**j**) and CBM (**k**,**l**) for monolayer SnS, VBM (**m**,**n**) and CBM (**o**,**p**) for monolayer SnSe illustrated in the bc and ac plane respectively. The isovalue is 0.0085 e/Å${}^{3}$.

According to the $d^{-2}$ principle [49], the inter-atomic overlap integral *V* determines the bandwidth of a specific band, and generally, a band with a larger bandwidth has a larger band curvature at the VBM/CBM points, which thus leads to smaller effective hole/electron masses for covalent solids from chemical intuition [50]. For the four MX monolayer under investigations here, the effective mass of holes $m_{h}^{*}$ is generally larger than that of electron $m_{e}^{*}$, which is due to the bonding character for VBM and antibonding character for CBM. The charge distributions for VBM/CBM of the four materials are shown in Figure 5. When applying tensile/compressive uniaxial strains, the overlap integral *V* will decrease/increase, especially along *x* direction due to the stronger π-bonding of *p* orbitals along *x* direction compared to *y* direction, as shown in Figure 5. Subsequently, the electron/hole effective masses along the *x* direction have the steeper tendency of increase under the uniaxial tensile strain compared to those along *y* direction.

As mentioned above, the locations of CBM/VBM may change when applying a uniaxial strain, and we used the chemical bonding theory to explain the underlying mechanism of the subsequent change of the effective masses of electrons/holes. The sharp change of VBM to the Γ point for GeS monolayer when applying strains larger than 2% along *x* or *y* direction, leads to the abrupt increase of effective mass of holes, as shown in Figure 4a,b. For GeSe monolayer, moderate changes around the original location of VBM take place when applying strains along *x* or *y* direction, which subsequently results in the continuous increase of effective mass of holes, as shown in Figure 4c,d. For SnS monolayer, locations of CBM/VBM do not change when applying strains along *y* direction, and the effective mass for both electrons and holes increase continuously, as shown in Figure 4f. Similar behavior can be seen in Figure 4h for the case of SnSe monolayer. However, when applying strains larger than 2% along *x* direction, the location of CBM ($p_{x}$ orbital dominates for both SnS and SnSe monolayers) changes to the position of CB1 ($p_{y}$ orbital dominates for both SnS and SnSe monolayers) as shown in Figure 2i, an abrupt change therefore appears for the curves of effective masses, as shown in Figure 4e. For SnSe monolayer, when applying strains larger than 1% along *x* direction, location of CBM changes abruptly to the position of CB1, the effective mass of electrons along *x* direction $m_{e}^{x}$ calculated at CB1 dominantly attributed by $p_{y}$ orbitals is larger than the effective mass of holes $m_{h}^{x}$ calculated at VBM dominantly attributed by $p_{x}$ orbitals, which is probably due to the much weaker bonding of $p_{y}$ orbitals in *x* direction compared to x-direction bonding by $p_{x}$ orbitals. Further increase of $m_{e}^{x}$ can be seen in Figure 4g when increasing tensile strains along *x* direction due to the decrease of *V*.

## 4. Conclusions

In summary, we have performed first-principles calculations on the orbital contributions band structures, angular dependence and strain engineering of monolayer group IV monochalcogenides MX (*M* = Ge, Sn; *X* = S, Se). With similar puckered atom structure to that of black phosphorene, monolayer GeS, SnS and SnS demonstrate indirect band gaps, and GeSe has direct semiconductor property. Compared to the other three group IV monochalcogenides, monolayer GeS has the largest hole effective mass ratio $\gamma_{h}$ of 3.664 and electron effective mass $\gamma_{e}$ of 2.588, showing strongly anisotropic property. For the hole effective mass along the *x* direction, it has an immense increase (from 0.361$m_{0}$ to 6.419$m_{0}$) under uniaxial strain of $\varepsilon_{x}$ = 5%, while under strain along the *y* direction, the values of carrier effective mass vary in a low mass range. The remarkable anisotropy existed in the 2D MX may provide another new degree of freedom for the design of microelectronic devices, and the property of easily tunable semiconductor type of monolayer GeSe and SnSe may lead to potential applications in optoelectronics.

## Figures and Tables

**Table 1:** The optimized lattice constants, bond length and bond angle for monolayer black phosphorene and phosphorene analogues (monolayer group IV monochalcogenides MX) (*M* = Ge, Sn; *X* = S, Se), respectively.

Structure	*a* (Å)	*b* (Å)	$\gamma_{\left(b/a\right)}$	$d_{1}$ (Å)	$d_{2}$ (Å)	$\theta_{1}$ (deg)	$\theta_{2}$ (deg)	$\theta_{3}$ (deg)
P	3.298	4.627	N.A.	2.259	2.220	95.917	104.182	104.182
GeS	3.620	4.492	1.241	2.411	2.452	95.165	95.239	104.123
GeSe	3.935	4.248	1.080	2.529	2.642	96.263	93.145	97.863
SnS	4.029	4.257	1.057	2.575	2.721	95.525	89.423	100.024
SnSe	4.248	4.346	1.023	2.706	2.892	94.525	93.559	92.504

**Table 2:** Semiconductor type, band gap calculated under PBE and HSE levels, energy difference ($E_{\Delta }$) between CBM and CB1, calculated carrier (holes and electrons) effective mass along the transport directions and the anisotropic ratio $\gamma_{h,e}$ for holes and electrons of monolayer MX.

Structure	Type	$E_{g}^{\left(\mathrm{PBE}/\mathrm{HSE}\right)}$ (eV)	$E_{\Delta }$ (eV)	$m_{h}^{x}\;\left(m_{0}\right)$	$m_{h}^{y}\;\left(m_{0}\right)$	$\gamma_{h}$	$m_{e}^{x}\;\left(m_{0}\right)$	$m_{e}^{y}\;\left(m_{0}\right)$	$\gamma_{e}$
GeS	indirect	1.713/2.75	0.4092	0.850 (0.92 [42])	0.232 (0.23 [42])	3.664	0.572 (0.50 [42])	0.221 (0.22 [42])	2.588
GeSe	direct	1.066/1.61	0.0119	0.361 (0.38 [35])	0.130 (0.13 [42])	2.375	0.300 (0.31 [35])	0.140 (0.14 [42])	2.143
SnS	indirect	1.349/1.50	0.1707	0.287 (0.27 [42])	0.172 (0.22 [42])	1.669	0.200 (0.20 [42])	0.193 (0.19 [42])	1.039
SnSe	indirect	0.790/0.98	0.0559	0.128 (0.14 [43])	0.108 (0.13 [43])	1.185	0.108 (0.13 [43])	0.127 (0.14 [43])	0.850

**Table 3:** Evolution range of effective mass of hole and electron under tensile uniaxial strain of monolayer MX.

Structure	$\Delta m_{h}^{x}\;\left(m_{0}\right)$	$\Delta m_{h}^{y}\;\left(m_{0}\right)$	$\Delta m_{e}^{x}\;\left(m_{0}\right)$	$\Delta m_{e}^{y}\;\left(m_{0}\right)$
GeS	0.850–3.610	0.232–0.580	0.209–0.769	0.221–0.274
GeSe	0.361–6.418	0.152–0.230	0.237–1.350	0.141–0.187
SnS	0.272–0.444	0.172–0.253	0.196–0.267	0.157–0.208
SnSe	0.128–0.306	0.108–0.149	0.108–0.331	0.102–0.158
